# The role of the habenula in drug addiction

**DOI:** 10.3389/fnhum.2014.00174

**Published:** 2014-03-28

**Authors:** Kenia M. Velasquez, David L. Molfese, Ramiro Salas

**Affiliations:** Department of Psychiatry, Baylor College of MedicineHouston, TX, USA

**Keywords:** habenula, addiction, dependence, tobacco, nicotine, withdrawal

## Abstract

Interest in the habenula has greatly increased in recent years. The habenula is a small brain structure located posterior to the thalamus and adjacent to the third ventricle. Despite its small size, the habenula can be divided into medial habenula (MHb) and lateral habenula (LHb) nuclei that are anatomically and transcriptionally distinct. The habenula receives inputs from the limbic system and basal ganglia primarily via the stria medullaris. The fasciculus retroflexus is the primary habenular output from the habenula to the midbrain and governs release of glutamate onto gabaergic cells in the rostromedial tegmental nucleus (RMTg) and onto the interpeduncular nucleus. The resulting GABA released from RMTg neurons inactivates dopaminergic cells in the ventral tegmental area/substantia nigra compacta. Through this process, the habenula controls dopamine levels in the striatum. Thus, the habenula plays a critical role in reward and reward-associated learning. The LHb also modulates serotonin levels and norepinephrine release, while the MHb modulates acetylcholine. The habenula is a critical crossroad that influences the brain’s response to pain, stress, anxiety, sleep, and reward. Dysfunction of the habenula has been linked to depression, schizophrenia, and the effects of drugs of abuse. This review focuses on the possible relationships between the habenula and drug abuse.

## THE HABENULA – GENERAL OVERVIEW

Historically, the habenula has been neglected in the scientific literature, yet this small brain structure has increasingly been studied by neuroscientists and psychologists interested in pain, stress, anxiety, sleep, reward, depression, schizophrenia, and drug addiction. Habenula is Latin for “little rein,” owing to this small structure’s shape and location near the pineal gland and third ventricle. The habenula is divided into two anatomically and transcriptionally distinct structures: the medial habenula (MHb) and lateral habenula (LHb; Klemm, 2004). The LHb plays a critical role in the brain’s response to reward and thus has been more extensively studied than the MHb (Matsumoto and Hikosaka, 2007). In addition, the LHb has been linked to major depression (Sartorius et al., 2010), while the MHb has been linked to the effects of nicotine (Salas et al., 2009; Fowler et al., 2011; Frahm et al., 2011).

In terms of white matter connectivity, the stria medullaris is the primary habenular input and the fasciculus retroflexus is the primary output. Through the stria medullaris, the habenula receives inputs from the septum, hippocampus, ventral pallidum, lateral hypothalamus, globus pallidus, and other basal ganglia structures. The septum is the main input to the MHb, while the remaining structures project mainly to the LHb (Klemm, 2004). While the MHb projects to the LHb, no connections from the LHb to the MHb have been described (Kim and Chang, 2005). Based on the input received, activity in the habenula is hypothesized to encode an organism reward state. If the reward state is positive, habenular activity is generally diminished. If the reward state is negative (e.g., during disappointing events) the habenular signal is enhanced. Information about reward state is then projected via the fasciculus retroflexus axon bundle to midbrain structures. The fasciculus retroflexus is divided into two concentric regions. The outer region originates in the LHb and projects to the rostromedial tegmental nucleus (RMTg). The RMTg, sometimes called the “tail” of the ventral tegmental area (VTA), is a small nucleus that contains mainly inhibitory GABAergic cells and ultimately controls activity in VTA/substantia nigra compacta (SNc), locus coeruleus and raphe nucleus. The inner region of the fasciculus retroflexus originates in the MHb and projects to the cholinoceptive interpeduncular nucleus (IPN; Herkenham and Nauta, 1977, 1979; Jhou et al., 2009a; **Table 1**).

**Table 1:** The function and anatomic location of brain structures associated with the habenula.

Structure	Anatomic location	Function	Reference
Medial habenula (MHb)	Above the thalamus at its posterior end close to the midline.	Modulates acetylcholine	Klemm (2004)
Lateral habenula (LHb)	Above the thalamus at its posterior end close to the midline, lateral to the MHb.	Associated with negative emotions.	Matsumoto and Hikosaka (2007)
Stria medullaris	Located on the medial side of the thalamus; it’s a bundle of fibers that run along the roof of the third ventricle to the thalamus and then terminates in the habenula.	Primary habenular input; projects to the lateral habenula receives inputs from the septum, hippocampus, ventral pallidum, lateral hypothalamus, globus pallidus, and other basal ganglia structure.	Klemm (2004)
Fasciculus retroflexus	Axon bundle divided into two concentric regions. Outer region originates in the lateral habenula and projects to the rostoromedial tegmental nucleus (RMTg). Inner region originates in the MHb and projects to the cholinergic IPN.	Primary habenular output; reward state is relayed to the midbrain via the FR.	Klemm (2004), Matsumoto and Hikosaka (2007)
Interpeduncular nucleus (IPN)	At the floor of the midbrain.	The MHb projects to the IPN. Main cholinergic center.	Klemm (2004)
Substantia nigra compacta (SNc)/ventral tegmental area (VTA)	Collection of neurons located in the midbrain.	Where dopaminergic neurons are located. Receive input from the LHb through the RMTg.	Matsumoto and Hikosaka (2007), Jhou et al. (2009a)
Rostromedial tegmental nucleus (RMTg)	A small nucleus that contains mainly inhibitory GABAergic cells, formerly called the “tail of the VTA.”	Receives input from the LHb and projects to midbrain dopamine neurons (VTA/SNc).	Jhou et al. (2009b)

Because of the role of the habenula in the modulation of dopamine, serotonin, norepinephrine, and acetylcholine, this region was the object of detailed study for a number of years (Herkenham and Nauta, 1977, 1979; Wang and Aghajanian, 1977; Cuello et al., 1978; Lisoprawski et al., 1980; Herkenham, 1981; Stern et al., 1981; Hamill et al., 1984). However, due to the small size of the habenula, the difficulty of doing non-invasive studies in humans, the lack of a clearly defined functional role (links to feeding behavior, pain sensitivity, anxiety, parental behavior, nicotine effects, and other behaviors were all demonstrated), and the lack of suitable pharmacological agents for habenula research, interest in the habenula began to taper off until 2007 when Matsumoto and Hikosaka published a seminal report on the habenula as a region associated with the signaling of negative prediction error events in the brain (Matsumoto and Hikosaka, 2007). The habenula was then linked to addiction through a series of rodent experiments and human genome-wide association studies (GWAS). These studies demonstrated that the nicotinic receptors expressed in the habenula are not only associated with tobacco addiction (Amos et al., 2008; Berrettini et al., 2008; Bierut et al., 2008; Thorgeirsson et al., 2008), but also with alcohol and cocaine addiction (Grucza et al., 2008; Wang et al., 2009). The dual role of the habenula in reward and addiction makes the structure an ideal target for understanding the effects of drugs of abuse on the brain. To date, most drug abuse-related research on the habenula has focused on nicotine, although the effects of cocaine, morphine, and other substances on the habenula have also been researched.

## PRECLINICAL WORK ON THE ROLE OF THE MEDIAL HABENULA ON NICOTINE EFFECTS

The creation of nicotinic acetylcholine receptor (nAChR) subunit mutant mouse lines, both null mice (Picciotto et al., 1998; Xu et al., 1999a,b; Franceschini et al., 2002; Cui et al., 2003; Salas et al., 2003a,b, 2004b, 2009; Kedmi et al., 2004; Maskos et al., 2005) and gain-of-function mice (Broide et al., 2002; Tapper et al., 2004; Fonck et al., 2005), has allowed for the characterization of the effects of nicotine on different nAChR subunits (**Table 2**). Through this research, the MHb has emerged as a mediator of nicotine withdrawal symptoms after chronic nicotine treatment. The α2, α5, and β4 subunits are involved in withdrawal (Salas et al., 2003b, 2004b, 2009) while the α3, α5, and β4 subunits are necessary for nicotine-induced hypolocomotion (or sedation) and nicotine-induced seizures (Salas et al., 2003a, 2004a). The α5 subunit is involved in nicotine intake: α5 KO mice self-administer significantly more nicotine than wild type mice, suggesting that the α5 subunit is necessary for the aversive effects of nicotine (Fowler et al., 2011). In addition, the activity balance between the α5 and the β4 subunits in the MHb has been shown to regulate aversion to nicotine (Frahm et al., 2011). Although, the molecular mechanisms mediating the effect of the MHb on nicotine withdrawal are not well understood, it was shown that the pacemaker activity of cholinergic (but not peptidergic) neurons in the MHb is critical for withdrawal (Gorlich et al., 2013). The β2 subunit (usually expressed in combination with the α4 subunit) has been widely considered “the addiction subunit” in that nicotine self-administration in mice is dependent on the presence of this subunit (Picciotto et al., 1998). However, β2 null mice exhibit normal nicotine withdrawal symptoms, suggesting that the effects of nicotine on β2-containing nAChRs are not necessary for the appearance of withdrawal symptoms in nicotine-treated mice (Salas et al., 2004b). The α2, α5, and β4 subunits have each been implicated in nicotine withdrawal and these subunits are expressed in a brain region-specific manner that highlights the MHb. The α5 subunit is expressed in the MHb, IPN, some cortical areas, VTA, and in area CA1 of the hippocampus. This subunit is an “accessory” subunit that becomes functional when combined with other α- and β- subunits (Hsu et al., 2013). In addition to being expressed in the habenula, the α3 and β4 subunits are expressed in the pineal gland and in mitral cells in the olfactory bulb; the α3 subunit is also expressed in the thalamus (Wada et al., 1989; Salas et al., 2003a, 2004a). Further evidence for a link between nicotine withdrawal and the habenula has been observed following injection of mecamylamine, a non-specific nAChR blocker, into the MHb of nicotine-treated mice. Mecamylamine injection precipitates somatic symptoms of withdrawal such as shakes and scratching, but only when injected into the MHb or IPN. In addition, β4, α5, and α2 knockout mice show no nicotine withdrawal effects following mecamylamine injections, suggesting that the MHb/IPN circuit, with high levels of β4, α5, and α2 subunit expression, is involved in the expression of behaviors related to nicotine withdrawal through neural mechanisms linked to these nAChRs (Salas et al., 2009). Consistent with a role for the α2 subunit in nicotine withdrawal, a recent study by Lotfipour et al. (2013) showed an increase in nicotine self-administration and withdrawal behavior (paw tremors, head shakes, backing, curls, grooming, scratching, chewing, cage scratching, head nodding, and jumping) in α2 -/- mice. In a separate series of studies, Stanley Glick and his team examined the drug 18-methoxycoronaridine (18-MC), an antagonist of β4-containing nAChRs. In rodents, microinjection of 18-MC into the MHb – but not in several other areas – blocks the effects of nicotine and cocaine (Glick et al., 2000, 2006; Maisonneuve and Glick, 2003; Taraschenko et al., 2007). In summary, the β2 subunit-containing nAChR expressed in the VTA/SNc and in many other areas is essential for reward-related dopamine release in the striatum and is important for nicotinic self-administration in mice (Picciotto et al., 1998; Maskos et al., 2005). However, this subunit is not necessarily involved in nicotine withdrawal. In contrast, β4 subunit-containing nAChRs expressed in the MHb and α2-containing nAChRs (highly expressed in the IPN) mediate the symptoms of nicotine withdrawal.

**Table 2:** The function and anatomic location of nicotinic acetylcholine receptors (nAChR) associated with the habenula.

Receptors/subunit	Anatomic location	Function	Reference
Nicotinic acetylcholine receptors (nAChR)	Ligand-gated ion channels formed by a pentameric arrangement of alpha and beta subunits to create distinct muscle and neuronal receptors.	Neuronal communication; converts neurotransmitter binding in to membrane electrical depolarization; binds the addictive drug nicotine.	Hogg et al. (2003)
α2	IPN, hippocampus.	Involved in withdrawal symptoms after chronic nicotine treatment.	Salas et al. (2009), Lotfipour et al. (2013)
α3	Pineal gland, mitral cells in the olfactory bulb, thalamus, MHb.	Necessary for nicotine-induced hypolocomotion (or sedation) and nicotine-induced seizures. Necessary for normal development of the peripheral nervous system.	Xu et al. (1999a)
α4	Hippocampus, cortex, MHb, IPN.	Necessary for tolerance, reward and sensitization.	Tapper et al. (2004), Salas et al. (2004a)
α5	Moderately expressed in the MHb. Also expressed in the IPN, VTA, cerebral cortex and in area CA1 of the hippocampus.	Involved in withdrawal symptoms after chronic nicotine treatment.	Fowler et al. (2011), Frahm et al. (2011), Salas et al. (2009), Morel et al. (2013), Bailey et al. (2010)
α6	VTA. Usually expressed with the β3 subunit.	Important for addiction, reward and dopaminergic transmission.	Wang et al. (2013), Brunzell (2012)
α7	Hippocampus, cortex	Forms homopentamers. May be involved in schizophrenia-related behaviors.	Freedman (2014), Young and Geyer (2013)
β2	Ubiquitously expressed, usually with the α4 subunit.	Essential for nicotine self-administration in mice.	Picciotto et al. (1998)
β3	MHb, VTA. Usually expressed with the α6 subunit.	Important for dopamine release, addiction, and reward.	Cui et al. (2003)
β4	MHb, pineal gland and mitral cells in the olfactory bulb.	Implicated in nicotine withdrawal.	Salas et al. (2004b)

## GENOME WIDE ASSOCIATION STUDIES IN HUMANS

Drug addiction has been linked to both environmental and genetic variables. Several studies have shown that susceptibility to drug addiction has a strong genetic component (Tsuang et al., 1998; Karkowski et al., 2000; Kendler et al., 2000). To discern the relative effects of genes and environment, a series of familial studies examined inheritance, twins, and adoption (Hall et al., 2013). These studies suggest a 30–50% genetic component to drug addiction. Many genetic studies were based on *a priori* hypotheses about specific gene targets in each population. While those early studies were met with several successes, bias introduced by testing only a limited number of genes was inevitable. The development of new tools for examining genetic relationships across the entire genome GWAS opened a new era in genetic studies. In GWAS, a large number of genetic variants [typically hundreds of thousands or even millions of single nucleotide polymorphisms (SNPs)] are tested for association with a specific trait, such as smoking susceptibility, alcoholism, or drug addiction. Large samples (usually in the thousands) of control and affected populations are compared using rigorous corrections for multiple comparisons. Increased expression of specific gene variants within the affected population can thus be associated with the disease or condition being studied. It is important to note that these are association studies and not studies of causality (Amos, 2007).

Genome-wide association study studies of tobacco addiction have produced highly consistent results across laboratories with variants in the α3, α5, and β4 nAChRs gene cluster associated with several measures of tobacco use and addiction (Amos et al., 2008; Berrettini et al., 2008; Bierut et al., 2008; Thorgeirsson et al., 2008). In particular, a significant link between the rs16969968 α5 SNP and smoking addiction was identified, and a meta-analysis yielded a significance of *p* < 10^-^^71^ (Bierut, 2011). Brain expression of the α3, α5, and β4 nAChRs is highly restricted; the MHb is the only region that co-expresses the mRNA for all three of these subunits at either very high (α3 and β4) or moderate (α5) levels. The IPN, the main output from the MHb, is a primary site of α5 and β4 nAChR subunit expression. Thus, GWAS data suggests that the habenula and associated regions expressing the α5 and β4 nAChR subunits may play a role in tobacco addiction. Interestingly, the same SNPs that are associated with the risk of tobacco use are also associated with cocaine abuse (Grucza et al., 2008) and alcohol abuse (Wang et al., 2009). This suggests that the mechanism of addiction for different drugs of abuse shares overlapping genetic factors and anatomical brain regions. Given the role of the habenula in modulating reward and disappointment, it is not surprising that genes controlling habenular activity are also associated with drugs of abuse (Quick et al., 1999).

## THE LATERAL HABENULA AND THE NEGATIVE PREDICTION ERROR

Drugs of abuse increase dopamine levels in the striatum. Since dopamine levels have been related to reward, it is believed that drug-induced increases in dopamine are important for the abuse potential of drugs (Di Chiara and Imperato, 1988). Activity of dopaminergic cells in the VTA/SNc is of critical importance for reward signaling and in addictive behaviors. Indeed, early work in primates showed that dopaminergic cells in the VTA/SNc are activated by oral delivery of sweet juice (Montague et al., 1996; Schultz et al., 1997). However, more recent experiments have shown that the role of dopamine in reward is more complex than simple stimulus-response. Monkeys that learn to associate juice reward with a predictive cue, such as a flash of light, shift the spike of dopaminergic activity to the cue. Following cue-juice association, the juice no longer induces dopaminergic activity, yet withholding of the expected juice reward causes dopamine levels to drop. This suggests that dopamine may actually signal reward prediction, rather than reward itself, and a negative prediction error or “disappointment” following lack of reward (Montague et al., 1996; Schultz et al., 1997). The decrease in dopaminergic activity is analogous to a negative prediction error and has been hypothesized to play a role in learning from mistakes. Only recently has the neural signal responsible for this drop in dopamine release been localized to the LHb (Matsumoto and Hikosaka, 2007). In their seminal 2007 work, Matsumoto and Hikosaka trained monkeys to perform lateral saccadic eye movements. A saccade to one side predicted juice delivery while saccades to the other side did not predict juice reward. By altering the probability of juice reward, Matsumoto and Hikosaka were able to create conditions of both unexpected reward and of reward disappointment when the juice was expected, but not delivered. They demonstrated that activity of neurons in the LHb increased following negative prediction error events (lack of expected reward) and decreased following unexpected juice reward. It follows that the habenula is activated by negative events. This activation decreases downstream dopaminergic activity through GABAergic inhibition of brain structures receiving habenular inputs and results in negative affect (Matsumoto and Hikosaka, 2007). In humans, the decreased reward experienced by chronic drug users may result from increased habenular activity and the resulting inhibition of dopamine in the VTA/SNc.

## HABENULAR CONTROL OF DOPAMINE, SEROTONIN, AND NOREPINEPHRINE

Neural signals from the LHb ultimately affect the VTA/SNc, the raphe nucleus, and the locus coeruleus (both directly and indirectly through the RMTg; Klemm, 2004; Balcita-Pedicino et al., 2011). The VTA/SNc is the most studied downstream habenular target, although both the raphe nucleus and locus coeruleus also receive strong signals from the LHb (Hikosaka et al., 2008). Given the role these structures play in the regulation of dopamine, serotonin, and norepinephrine, the LHb likely modulates neurotransmitter release in those three systems. Thus, it is not entirely surprising that habenular activity has been linked to several seemingly unrelated behaviors and disease conditions. For example, the habenula has been linked to stress-enhanced acoustic startle, probably via effects on the locus coeruleus (Heldt and Ressler, 2006). The LHb has been linked to maternal feeding and sexual behaviors (Klemm, 2004). The LHb has also been linked to major depressive disorder (MDD) in both preclinical and clinical research. In mice, learned helplessness (an animal model for studying depression-like symptoms) results in potentiation of LHb outputs, suggesting an increase in negative neural signaling (Li et al., 2011). Using cytochrome oxidase histochemistry, a metabolic marker, both the MHb and the LHb have been implicated in learned helplessness and depressive behavior in rats reared to exhibit these traits (Shumake et al., 2004). In humans, the severity of depression symptoms in patients correlated with the level of coupling between the habenula and the raphe nucleus: The stronger the link between these brain structures, the more severe the depression symptoms after a tryptophane-free diet was given to depression patients in remission to acutely precipitate depression symptoms (Morris et al., 1999). The link between habenular activity and MDD was further demonstrated in a treatment-resistant MDD patient who responded to deep brain stimulation of the LHb when other treatments for depression were shown to be ineffective (Sartorius et al., 2010). Taken together, these findings suggest that MDD may result from a combination of habenular hyperactivity that disrupts dopaminergic and serotonergic signaling following negative or disappointing events along with decreased dopaminergic activity following positive events.

## THE ROLE OF THE HABENULA IN COCAINE DEPENDENCE

Using cocaine reinstatement protocols it was demonstrated that high reinstatement rats and mice show higher c-fos activity than low reinstatement animals (Brown et al., 2010; James et al., 2011). While cocaine is initially rewarding, cocaine aversion usually develops about 15 min after injection (Ettenberg et al., 1999). Signals from the LHb inhibit dopaminergic neurons reducing the dopaminergic reward signal which can account for the aversive effects of cocaine (Jhou et al., 2013).

A study by Maroteaux and Mameli (2012) showed the synaptic plasticity of LHb neurons in mice, when exposed to cocaine. They demonstrated that exposure to cocaine selectively potentiates alpha-amino-3-hydroxy-5-methyl-4-isoxazolepropionic acid receptor (AMPAR)-mediated transmission in LHb neurons projecting to the RMTg but not to the VTA. They stereotaxically injected green and red fluorobeads in the VTA and RMTg and then examined if cocaine affected excitatory synaptic transmission on the LHb neurons. These mice were injected with cocaine and saline for two consecutive days. They found that cocaine exposure strengthens AMPAR-mediated synaptic transmission but only in LHb to RMTg neurons. Their data suggests that the composition of the synaptic AMPARs in LHb to RMTg neurons was not significantly changed by the cocaine. A residual current at positive potentials was detected, suggesting that excitatory synapses onto LHb neurons likely contain GluA2-lacking and GluA2-containing AMPARs. In sum, they offer evidence for a synaptic potentiation in the LHb that could represent an important mechanism involved in the formation of drug-associated memories (Maroteaux and Mameli, 2012).

## THE ROLE OF THE HABENULA IN MORPHINE DEPENDENCE

The habenula has also been implicated in morphine addiction, although the exact relationship between habenular activity and morphine is inconsistent across studies. Chronic morphine administration (escalating doses of 20, 40, 60, 80, 100, and 100 mg/kg three times per day) leads to a reduction in acetylcholinesterase (AChE) activity in the MHb (Neugebauer et al., 2013). During morphine withdrawal, AChE activity in MHb returns to baseline, suggesting a homeostatic balance. However, a second study using higher doses of morphine (450 mg/kg) administered for a longer period of time (15 days), showed increased AChE activity in the habenula (Mohanakumar and Sood, 1983).

Unlike cocaine, which increases Fos protein expression in the habenula (Brown et al., 2010; James et al., 2011), chronic morphine exposure reduces c-fos activity in the MHb (Neugebauer et al., 2013). Morphine withdrawal has also been shown to increase glucose metabolism in a number of limbic and thalamic regions, including the MHb (Kimes et al., 1990). Again suggestive of a homeostatic balancing mechanism, morphine withdrawal elevates Fos protein levels in the LHb. Somewhat surprisingly, similar patterns of c-fos positive cell activation have been reported in animals seeking sucrose following abstinence (Madsen et al., 2012).

## THE FASCICULUS RETROFLEXUS DEGENERATES FOLLOWING TREATMENT WITH DRUGS OF ABUSE

The rodent fasciculus retroflexus has been shown to degenerate following chronic exposure to stimulants such as D-amphetamine, methamphetamine, MDMA, cocaine, and nicotine. The fasciculus retroflexus nerve bundle connects the habenula to its main targets, the IPN and RMTg. Interestingly, nicotine treatment prompts cell death and axonal degeneration in the central region of the fasciculus (axons coming from the MHb) while other drugs of abuse prompted neurodegeneration in the external region (axons coming from the LHb; Ellison, 2002; Ciani et al., 2005). A recent study of fasciculus retroflexus integrity in rats showed neurodegeneration of the habenular efferent following increased cocaine self-administration, indicating a reduction in LHb to midbrain connectivity (Lax et al., 2013). Overall, the fasciculus retroflexus appears to be a “weak link” in the brain’s reward circuitry, one that is particularly susceptible to drugs of abuse (Ellison, 2002). The cause of fasciculus retroflexus degeneration remains unclear, however, multiple drugs of abuse may affect this pathway in that the habenula is activated during cocaine, morphine, and nicotine drug seeking behavior and during drug re-instatement following abstinence (Brown et al., 2010; James et al., 2011).

## 18-MC, AN ANTAGONIST OF β4-CONTAINING nAChR, DECREASES SEVERAL EFFECTS OF ABUSED DRUGS

Ibogaine, an extract from the African shrub Tabermanthe iboga, is a hallucinatory alkaloid used in African spiritual ceremonies. In the 1960s, ibogaine was used to treat several forms of addiction through apparent effects on the cholinergic and glutamatergic neurotransmitter systems. In rodents, ibogaine was shown to reduce self-administration of cocaine (Cappendijk and Dzoljic, 1993), alcohol (Rezvani et al., 1995), and morphine (Glick et al., 1991). However, because of the severe side-effects associated with ibogaine (e.g., headaches, nausea, hallucinations), the alkaloid has been banned in the USA and other countries. Ibogaine drug derivatives, such as 18-MC, however, have been created in an attempt to develop medications to treat drug addiction. The 18-MC compound has been shown to effectively block cravings and withdrawal and to decrease self-administration of morphine, cocaine, and nicotine in laboratory animals (Glick et al., 2000). The drug does not affect water consumption, unlike the naturally occurring Ibogaine. Furthermore, the effectiveness of 18-MC increases with repeated treatments and reduces the overall intensity of morphine withdrawal symptoms in rats (Glick et al., 2000). The mechanism of action for 18-MC and the site of action remain unclear. While direct injection of 18-MC into the locus coeruleus reduces symptoms of morphine withdrawal, direct injection into the MHb has mixed effects. Low doses of 18-MC in the habenula reduce teeth chattering and weight loss while higher doses increase teeth chattering and reduce burying behaviors during morphine withdrawal (Panchal et al., 2005). 18-MC also blocks the acquisition of conditioned place preference (CPP) for cocaine, but enhances the reinstatement of cocaine-seeking behavior following extinction (McCallum and Glick, 2009). 18-MC does not affect glutamate receptors, but retains antagonistic activity toward the highly expressed β4-containing nAChRs in the MHb and IPN (Glick et al., 2006). Additionally, 18-MC affects dopaminergic activity, which is regulated by the LHb. Consequently, targeting of β4-containing nAChRs in the MHb could prove therapeutic to the treatment of nicotine, cocaine, morphine, alcohol, and methamphetamine addiction.

## VARENICLINE AFFECTS DEPENDENCE ON DRUGS OF ABUSE

Varenicline is a partial agonist of nAChRs and is approved by the FDA for use in tobacco quitting therapies (Coe et al., 2005). The effects of varenicline on tobacco addiction are well studied, but it is becoming evident that varenicline may also be effective in the treatment of other drugs of abuse. Varenicline effectively weakens cocaine reinstatement in small doses, but increases cocaine reinstatement in higher doses even as it decreases self-administration (Guillem and Peoples, 2010). In rodent studies of varenicline and alcohol abuse, the drug reduced ethanol seeking behavior at the same doses previously shown to reduce nicotine-seeking behavior, but did not alter sucrose-seeking behavior in self-administration experiments. In mice, voluntary ethanol consumption was also decreased, but not water consumption (Steensland et al., 2007). These results suggest that varenicline may be an effective treatment not only for tobacco but also for other drugs of abuse (Crunelle et al., 2010). Although most of the literature points to α4β2-containing nAChR as the relevant target the α3β4 subunit receptor has also been implicated in addiction and the β4 subunit was recently linked to varenicline’s reduction of ethanol consumption (Chatterjee et al., 2011). Mihalak et al. (2006) have shown that varenicline is a partial agonist of both α4β2 and α3β4 nAChRs. Thus, it is likely that at least some of the effects of varenicline on drug abuse are mediated by the habenula where these receptors are expressed at high concentration.

## THE MEDIAL AND THE LATERAL HABENULA

The role of the MHb in drug addiction and abuse has been demonstrated through rodent studies of nicotinic receptors as well as suggested by GWAS studies in humans. A role for the LHb in addiction is suggested by negative prediction error studies (Matsumoto and Hikosaka, 2007; Salas et al., 2010), deep brain stimulation in rodents (Lax et al., 2013), and drug-induced fasciculus retroflexus degeneration in rodents (Ellison, 1992, 2002). While it is possible that the MHb and the LHb regulate distinct mechanisms of drug dependence and abuse, we believe that this is not necessarily the case. McCallum et al. (2012) recently showed that blockade of β4 subunit-containing nAChRs in the MHb directly affects mesolimbic dopamine. Microinjections of 18-MC or AuIB (both preferential β4 antagonists) into the MHb prevented nicotine-induced increases in nucleus accumbens (NAcc) dopamine. Thus, there must be a connection between the MHb and NAcc dopamine levels (McCallum et al., 2012). The MHb may regulate dopamine in striatal regions through several mechanisms, although two candidate pathways appear most likely. First, the IPN is the primary target of the MHb and projects directly to dopaminergic areas, making it possible that activity in the MHb influences dopamine levels in the striatum independent of LHb activity (Groenewegen et al., 1986; Klemm, 2004). Second, direct connections from the MHb to the LHb have also been reported, but not in the opposite direction, making it possible that one of the functions of the MHb is to provide additional input to the LHb (Kim and Chang, 2005). The mechanisms by which these structures may modulate dopamine availability in the striatum are unknown at this time.

## ELECTRICAL STIMULATION IN THE LATERAL HABENULA REDUCES COCAINE-SEEKING BEHAVIOR

Deep brain stimulation is an effective treatment for several neurological diseases and conditions, including Parkinson’s disease and depression (Mayberg, 2009; Callesen et al., 2013). In rodent studies, electrical stimulation of the LHb reduced cocaine cravings, self-administration, and reinstatement while improving extinction. Damage to the LHb increased cocaine-seeking behavior, suggesting that this behavior may result from a weakening of cocaine-induced glutamatergic inputs to the VTA (Friedman et al., 2010). Since cocaine treatment causes degeneration of the fasciculus retroflexus (Ellison, 1992), it is important to know the status of the fasciculus retroflexus in human cocaine users before attempting to use deep brain stimulation as a possible therapy for cocaine abuse.

## INSIGHT FROM HUMAN fMRI STUDIES

Most of what we know about the habenula comes from rodent and monkey models. In humans, study of the habenula has been hampered by its small size. In the first functional magnetic resonance imaging (fMRI) report focusing on the habenula, the authors asked human participants to guess which of two visual stimuli would reach a target first. The task difficulty was modulated so that all participants would commit a similar number of errors and receive the same degree of positive and negative feedback. Negative feedback activated the habenula (Ullsperger and von Cramon, 2003). Using a similar strategy, Shepard et al. (2006) reported that schizophrenic patients lack habenular activation following negative feedback. Of the limited number of human fMRI studies focusing on the habenula, only one specifically addressed reward. In that study, a juice reward paradigm previously used in monkey studies of the habenula was employed. Juice was used as a reward and delay of expected juice was used as a disappointing stimulus. As in the monkey studies, the human habenula is activated following non-delivery of expected juice reward (Salas et al., 2010). A related study by Ide and Li (2011) used fMRI connectivity analysis to study humans performing a stop signal task in which subjects had to rapidly follow the instruction of “go” or “stop.” Each “stop” event could be successful (the prompt called for a “stop”) or failed (the prompt indicated “go” when the participant stopped). The investigators looked for regions with larger psychophysiological interaction with the habenula during stop-error than during stop-success trials and identified task-specific activation of the VTA/SNc, internal segment of globus pallidus, bilateral amygdala, and insula. Using Granger causality and mediation analyses they showed a directional link between habenula and VTA/SNc fMRI activation. These results suggest that habenular activity results in a decrease in VTA/SNc activity (Ide and Li, 2011). Finally, there are two reports linking the habenula to human MDD. In a PET study, Morris et al. (1999) observed increased coupling between habenula/raphe nucleuses following tryptophan depletion in former MDD patients exhibiting signs of remission. This increase in habenular/raphe coupling correlated with the re-appearance of depression symptoms (Morris et al., 1999). Anatomically, Savitz et al. (2011) presented evidence for a reduction in habenular volume in non-medicated, depressed bipolar disorder patients and female MDD patients.

## CONCLUSION

Much of the work linking the habenula to drug addiction is based on studies of nicotine and nicotinic receptors in the MHb. Additional studies have linked the habenula to morphine and cocaine addiction and withdrawal. Importantly, deep brain stimulation of the habenula has been shown to decrease cocaine-seeking behavior in rodents, which may open the door to possible therapies against cocaine abuse. A caveat to this approach is that cocaine and other drugs of abuse are known to cause degeneration of the fasciculus retroflexus, the primary habenular output, potentially reducing the effectiveness of deep brain stimulation as a treatment for drug abuse. Similarly, nicotine causes degeneration of the internal component of the fasciculus retroflexus, which connects the MHb to the IPN. As a result of this “weak link” between the habenula and downstream structures, the IPN and the RMTg may be more effective primary targets for anti-addiction drugs. Additional research is required to better understand the role of the MHb and the LHb in drug addiction and to illuminate the relationship between the habenula and downstream structures in the face of addiction. Promising technological advances, such as the use of 7T MRI scanners, may soon allow researchers to differentiate the roles of the MHb and the LHb in humans with drug addiction, depression, and other disorders (Strotmann et al., 2013). The habenula is a worthy target for study in that it has been shown to play a role in addiction, depression, and negative feedback and negative prediction error events (likely two manifestations of the same phenomenon). The habenula also acts as a major regulator of several neurotransmitter systems, including dopamine, serotonin, norepinephrine, and acetylcholine. These neurotransmitters alter neural activity throughout the brain and thus have the potential to influence a wide-range of both normal and abnormal human behaviors.

## Conflict of Interest Statement

The authors declare that the research was conducted in the absence of any commercial or financial relationships that could be construed as a potential conflict of interest.

## Abbreviations

IPN: interpeduncular nucleus
VTA: ventral tegmental area
RMTg: rostromedial tegmental nucleus
nAChR: nicotinic acetylcholine receptor
GWAS: genome-wide association study
SNP: single nucleotide polymorphism
fMRI: functional magnetic resonance imaging
SNc: substantia nigra compacta
